# Supplementary material for: Establishing the measurement and psychometrics of medical student feedback literacy (IMPROVE-FL): A research protocol
Source: PLoS One. 2024 Nov 7;19(11):e0313332. doi: 10.1371/journal.pone.0313332 (PMC11542893; doi:10.1371/journal.pone.0313332)
Supplement: S1 Appendix — (DOCX) [file pone.0313332.s001.docx]

**S1 Appendix**

**Full search string of each database**

1. Medline

( medic* OR health OR "health science*" OR clinic* OR nurs* OR biomedic* OR pharma* OR nutrition OR dietetic* OR dental OR dentist* OR "allied health" OR "occupational health" OR "environmental health" OR "occupational therap*" OR physiotherap* OR "phsyical therap*" OR "speech therap*" OR "speech language phatolog*" OR "occupational safety" OR psycholog* OR audiolog* OR forensic* OR radiotherap* ) AND ( "feedback literacy" OR "feedback recepti*" OR "feedback receiv*" OR "receiving feedback" OR "feedback orientation" OR "feedback perception" OR "perception of feedback" OR "feedback conception" OR "feedback seeking" OR "feedback behavio*" OR "feedback attitude" OR "feedback culture" OR "feedback dialogue" OR "feedback acceptance" OR "perspectives of feedback" OR "feedback perspectives" OR "feedback practice" ) AND ( validit* OR reliabilit* OR sensitivit* OR precision OR specificit* OR responsiveness OR psychometri* OR "coefficient of variation" OR "cognitive interview" OR comprehensi* OR "factor analysis" OR "internal consistenc*" OR "reproducibilit*" OR Cronbach OR "structural equation model*" OR "measurement invariance" ) AND ( instrument* OR measur* OR test OR assessment OR evaluat* OR tool OR questionnaire OR survey )

Expanders: Apply related words, Apply equivalent subjects

Limiters: English Language, Human, Language: English, Scholarly (Peer Reviewed) Journals

1. Scopus

( TITLE-ABS-KEY ( medic* OR health OR "health science*" OR clinic* OR nurs* OR biomedic* OR pharma* OR nutrition OR dietetic* OR dental OR dentist* OR "allied health" OR "occupational health" OR "environmental health" OR "occupational therap*" OR physiotherap* OR "phsyical therap*" OR "speech therap*" OR "speech language phatolog*" OR "occupational safety" OR psycholog* OR audiolog* OR forensic* OR radiotherap* ) AND TITLE-ABS-KEY ( "feedback literacy" OR "feedback recepti*" OR "feedback receiv*" OR "receiving feedback" OR "feedback orientation" OR "feedback perception" OR "perception of feedback" OR "feedback conception" OR "feedback seeking" OR "feedback behavio*" OR "feedback attitude" OR "feedback culture" OR "feedback dialogue" OR "feedback acceptance" OR "perspectives of feedback" OR "feedback perspectives" OR "feedback practice" ) AND TITLE-ABS-KEY ( validit* OR reliabilit* OR sensitivit* OR precision OR specificit* OR responsiveness OR psychometri* OR "coefficient of variation" OR "cognitive interview" OR comprehensi* OR "factor analysis" OR "internal consistenc*" OR "reproducibilit*" OR cronbach OR "structural equation model*" OR "measurement invariance" ) AND TITLE-ABS-KEY ( instrument* OR measur* OR test OR assessment OR evaluat* OR tool OR questionnaire OR survey ) ) AND ( LIMIT-TO ( DOCTYPE , "ar" ) ) AND ( LIMIT-TO ( LANGUAGE , "English" ) ) AND ( LIMIT-TO ( SRCTYPE , "j" ) )

1. Web of Science

(((TS=(medic* OR health OR "health science*" OR clinic* OR nurs* OR biomedic* OR pharma* OR nutrition OR dietetic* OR dental OR dentist* OR "allied health" OR "occupational health" OR "environmental health" OR "occupational therap*" OR physiotherap* OR "phsyical therap*" OR "speech therap*" OR "speech language phatolog*" OR "occupational safety" OR psycholog* OR audiolog* OR forensic* OR radiotherap*)) AND TS=("feedback literacy" OR "feedback recepti*" OR "feedback receiv*" OR "receiving feedback" OR "feedback orientation" OR "feedback perception" OR "perception of feedback" OR "feedback conception" OR "feedback seeking" OR "feedback behavio*" OR "feedback attitude" OR "feedback culture" OR "feedback dialogue" OR "feedback acceptance" OR "perspectives of feedback" OR "feedback perspectives" OR "feedback practice")) AND TS=(validit* OR reliabilit* OR sensitivit* OR precision OR specificit* OR responsiveness OR psychometri* OR "coefficient of variation" OR "cognitive interview" OR comprehensi* OR "factor analysis" OR "internal consistenc*" OR "reproducibilit*" OR Cronbach OR "structural equation model*" OR "measurement invariance")) AND TS=(instrument* OR measur* OR test OR assessment OR evaluat* OR tool OR questionnaire OR survey) and Preprint Citation Index (Exclude – Database) and Article (Document Types) and English (Languages)

1. CINAHL Complete

TX ( medic* OR health OR "health science*" OR clinic* OR nurs* OR biomedic* OR pharma* OR nutrition OR dietetic* OR dental OR dentist* OR "allied health" OR "occupational health" OR "environmental health" OR "occupational therap*" OR physiotherap* OR "phsyical therap*" OR "speech therap*" OR "speech language phatolog*" OR "occupational safety" OR psycholog* OR audiolog* OR forensic* OR radiotherap* ) AND TX ( "feedback literacy" OR "feedback recepti*" OR "feedback receiv*" OR "receiving feedback" OR "feedback orientation" OR "feedback perception" OR "perception of feedback" OR "feedback conception" OR "feedback seeking" OR "feedback behavio*" OR "feedback attitude" OR "feedback culture" OR "feedback dialogue" OR "feedback acceptance" OR "perspectives of feedback" OR "feedback perspectives" OR "feedback practice" ) AND TX ( validit* OR reliabilit* OR sensitivit* OR precision OR specificit* OR responsiveness OR psychometri* OR "coefficient of variation" OR "cognitive interview" OR comprehensi* OR "factor analysis" OR "internal consistenc*" OR "reproducibilit*" OR Cronbach OR "structural equation model*" OR "measurement invariance" ) AND TX ( instrument* OR measur* OR test OR assessment OR evaluat* OR tool OR questionnaire OR survey )

Expanders; Apply related words, Apply equivalent subjects

Limiters; English Language, Peer Reviewed, Research Article, Language: English, Publication Type: Journal Article

1. Education Research Complete

( medic* OR health OR "health science*" OR clinic* OR nurs* OR biomedic* OR pharma* OR nutrition OR dietetic* OR dental OR dentist* OR "allied health" OR "occupational health" OR "environmental health" OR "occupational therap*" OR physiotherap* OR "phsyical therap*" OR "speech therap*" OR "speech language phatolog*" OR "occupational safety" OR psycholog* OR audiolog* OR forensic* OR radiotherap* ) AND ( "feedback literacy" OR "feedback recepti*" OR "feedback receiv*" OR "receiving feedback" OR "feedback orientation" OR "feedback perception" OR "perception of feedback" OR "feedback conception" OR "feedback seeking" OR "feedback behavio*" OR "feedback attitude" OR "feedback culture" OR "feedback dialogue" OR "feedback acceptance" OR "perspectives of feedback" OR "feedback perspectives" OR "feedback practice" ) AND ( validit* OR reliabilit* OR sensitivit* OR precision OR specificit* OR responsiveness OR psychometri* OR "coefficient of variation" OR "cognitive interview" OR comprehensi* OR "factor analysis" OR "internal consistenc*" OR "reproducibilit*" OR Cronbach OR "structural equation model*" OR "measurement invariance" ) AND ( instrument* OR measur* OR test OR assessment OR evaluat* OR tool OR questionnaire OR survey )

Expanders: Apply equivalent subjects, Apply related words

Limiters: Peer Reviewed, Publication Type: Academic Journal, Document Type: Article, Language: English

1. Psychology and Behavioural Sciences Collection

( medic* OR health OR "health science*" OR clinic* OR nurs* OR biomedic* OR pharma* OR nutrition OR dietetic* OR dental OR dentist* OR "allied health" OR "occupational health" OR "environmental health" OR "occupational therap*" OR physiotherap* OR "phsyical therap*" OR "speech therap*" OR "speech language phatolog*" OR "occupational safety" OR psycholog* OR audiolog* OR forensic* OR radiotherap* ) AND ( "feedback literacy" OR "feedback recepti*" OR "feedback receiv*" OR "receiving feedback" OR "feedback orientation" OR "feedback perception" OR "perception of feedback" OR "feedback conception" OR "feedback seeking" OR "feedback behavio*" OR "feedback attitude" OR "feedback culture" OR "feedback dialogue" OR "feedback acceptance" OR "perspectives of feedback" OR "feedback perspectives" OR "feedback practice" ) AND ( validit* OR reliabilit* OR sensitivit* OR precision OR specificit* OR responsiveness OR psychometri* OR "coefficient of variation" OR "cognitive interview" OR comprehensi* OR "factor analysis" OR "internal consistenc*" OR "reproducibilit*" OR Cronbach OR "structural equation model*" OR "measurement invariance" ) AND ( instrument* OR measur* OR test OR assessment OR evaluat* OR tool OR questionnaire OR survey )

Expanders: Apply equivalent subjects, Apply related words

Limiters: English Language, Peer Reviewed, Document Type: Article
