# Supplementary material for: Establishing the measurement and psychometrics of medical student feedback literacy (IMPROVE-FL): A research protocol
Source: PLoS One. 2024 Nov 7;19(11):e0313332. doi: 10.1371/journal.pone.0313332 (PMC11542893; doi:10.1371/journal.pone.0313332)
Supplement: Attachment — Submitted filename: Response to Reviewers v4.docx [file pone.0313332.s002.docx]

| **EDITOR COMMENTS** | |
| --- | --- |
| Comments | Response |
| The protocol of coding is not clear, and it has not explained in detail, it affected the thematic realisations. | Thank you for your comment. We appreciate your efforts in providing feedback to our manuscript and helping us to improve on it. We have amended sub-section “Thematic analysis” as per your suggestion.  **Thematic analysis**  Interview transcripts will be thematically analyzed using NVivo, as this approach is flexible and accessible [47]. As an example, medical students, educators, and patients will be asked the prompt, “Please describe how medical students should receive feedback;” hence, the triangulation of their experiences may provide a comprehensive description of medical student feedback literacy. Codes will be developed based on the collected data [30, 47]. This inductive approach allows for a novel and comprehensive description of medical students’ feedback literacy, that is not confined to existing concepts. The researcher will read the interview transcripts multiple times to become familiar with them. Recurring phrases or keywords will be marked to identify possible patterns in the data and cross-check the patterns that appear among medical students, educators, and patients. Subsequently, codes will be generated to capture notable patterns, which will be checked to reduce overlap and redundancy, and subsequently grouped into potential themes, using an iterative to refine each theme. These themes will be named and clearly defined. Next, these themes will be examined to the entire dataset to ensure that the conclusions made are firmly rooted in the data. The themes will be organized and presented as a thematic map. Additionally, researchers (other than the analyst) will review the themes to enhance the credibility of thematic analysis findings [48]. Finally, the themes will be analyzed to generate measurable domains of medical students’ feedback literacy. |
| The reliability of analysis and coding identification need to be clarified and explained. | We appreciate the insightful feedback. If reliability refers to the consistency of findings from analysis and coding identification, we have employed the methods listed below. Upon reflection and reviewer comments, we identified that there is a room for improvement, and this triggered us to conduct reviews for the codes and themes developed, to ensure credibility and consistency of our analysis.   1. Each transcript is returned to respective participants for member-checking. 2. Interview data is collected from three types of informants; medical students, medical educators, and patients to allow data triangulation. 3. Reviews will be conducted by other researchers (different from the analyst) to enhance credibility of the thematic analysis results . 4. The themes will be examined to the raw data to ensure accurate representation of the data.   **Interviews with medical students, medical educators, and patients**  Interviews will be conducted by researchers specifically trained for this purpose. The interview sessions will be audiotaped and then transcribed verbatim for further analysis. The transcripts of the interviews will be returned to the interviewees for their own review to ensure accuracy with their experiences (member-checking) [30].  **Thematic analysis**  Interview transcripts will be thematically analyzed using NVivo, as this approach is flexible and accessible [47]. As an example, medical students, educators, and patients will be asked the prompt, “Please describe how medical students should receive feedback;” hence, the triangulation of their experiences may provide a comprehensive description of medical student feedback literacy. Codes will be developed based on the collected data [30, 47]. This inductive approach allows for a novel and comprehensive description of medical students’ feedback literacy, that is not confined to existing concepts. The researcher will read the interview transcripts multiple times to become familiar with them. Recurring phrases or keywords will be marked to identify possible patterns in the data and cross-check the patterns that appear among medical students, educators, and patients. Subsequently, codes will be generated to capture notable patterns, which will be checked to reduce overlap and redundancy, and subsequently grouped into potential themes, using an iterative to refine each theme. These themes will be named and clearly defined. Next, these themes will be examined to the entire dataset to ensure that the conclusions made are firmly rooted in the data. The themes will be organized and presented as a thematic map. Additionally, researchers (other than the analyst) will review the themes to enhance the credibility of thematic analysis findings [48]. Finally, the themes will be analyzed to generate measurable domains of medical students’ feedback literacy. |
| The instrument validation was not considered. | We appreciate the insightful feedback. To clarify, we have described our plans to validate the instrument, and these can be referred to section “Phase 3: Validating the measurable domains of Medical Student Feedback Literacy”, from sub-section “Items generation” to “Cross-cultural validity”. In addition, we have amended the third paragraph of the Discussion section to enhance clarity on the validation steps that will be employed for our future instrument.  **Discussion**  The methodology presented in this protocol has several strengths. This research study can explore feedback literacy in medical education to create a consolidated definition. Each step of the project will be guided by validated, frequently used methods for producing an evidence-based research protocols. The instrument will undergo multiple procedures to ensure its validity and reliability, including expert validation and cognitive interviews for content validity, exploratory and confirmatory analyses for construct, convergent and discriminant validities, calculation of Cronbach’s alpha for internal consistency, hypothesis testing with the Implicit Theory of Intelligence Scale for construct validity, test-retest reliability, and international surveys for cross-cultural validity. Implementation of this protocol should result in a comprehensive and accurate measurement of medical students’ feedback literacy. |
| The paper needs proofreading. | We have sent the manuscript to Editage for language edit and proofreading, and the certificate of language edit is attached. |
| Please ensure that your manuscript meets PLOS ONE's style requirements, including those for file naming. | We have double-checked the manuscript and sent the manuscript to Editage for language edit and formatting, and the certificate of language edit is attached. |
| Please state what role the funders took in the study. | We have included the following statement in the cover letter.  *The funders had no role in study design, data collection and analysis, decision to publish, or preparation of the manuscript.* |
| If there are ethical or legal restrictions on sharing a de-identified data set, please explain them in detail (e.g., data contain potentially identifying or sensitive patient information, data are owned by a third-party organization, etc.) and who has imposed them (e.g., a Research Ethics Committee or Institutional Review Board, etc.). Please also provide contact information for a data access committee, ethics committee, or other institutional body to which data requests may be sent.  If there are no restrictions, please upload the minimal anonymized data set necessary to replicate your study findings to a stable, public repository and provide us with the relevant URLs, DOIs, or accession numbers. For a list of recommended repositories, please see <https://journals.plos.org/plosone/s/recommended-repositories>. You also have the option of uploading the data as Supporting Information files, but we would recommend depositing data directly to a data repository if possible. | To clarify, our manuscript is a study protocol with no preliminary or pilot study findings. We have not generated any data and the data will only be available once data collection and analysis is done. This has been reflected in our submission form, where we have stated “N/A – No results are reported” and “No datasets were generated or analysed during the current study. All relevant data from this study will be made available upon study completion” in the data availability section. We plan to store our data in the Figshare repository once the study is complete. |
| While revising your submission, please upload your figure files to the Preflight Analysis and Conversion Engine (PACE) digital diagnostic tool, <https://pacev2.apexcovantage.com/>. | We have uploaded the figure files to PACE as per your request. |

| **REVIEWER COMMENTS** | | |
| --- | --- | --- |
| Q | Comments | Response |
| 1. | *Does the manuscript provide a valid rationale for the proposed study, with clearly identified and justified research questions?*  **Yes.** | We thank the reviewer for all their comments. We appreciate your efforts in providing feedback to our manuscript and helping us to improve on it. |
| 2. | *Is the protocol technically sound and planned in a manner that will lead to a meaningful outcome and allow testing the stated hypotheses?*  **Yes.** | We thank the reviewer for their encouraging comment. |
| 3. | *Is the methodology feasible and described in sufficient detail to allow the work to be replicable?*  **Yes.** | We thank the reviewer for their encouraging comment. |
| 4. | *Have the authors described where all data underlying the findings will be made available when the study is complete?*  **No.** | To clarify, our manuscript is a study protocol with no preliminary or pilot study findings. We have not generated any data and the data will only be available once data collection and analysis is done. We plan to store our data in the Figshare repository once the study is complete. |
| 5. | *Is the manuscript presented in an intelligible fashion and written in standard English?*  **No.** | We have sent the manuscript to Editage for language edit, and the certificate of language edit is attached. |
| 6. | i. I recommend authors to review both teacher feedback literacy and student feedback literacy as they are quite different. Moreover, the interplay between these two concepts also deserves several sentences to discuss. | We appreciate the insightful feedback. We have amended the second and third paragraphs of the introduction section as per your suggestion.  **Introduction**  Current feedback models encourage learner autonomy. Learners must positively receive feedback, and teachers need to formulate and deliver feedback efficiently [7]. This shared responsibility motivates students to seek, process and respond to feedback from multiple sources and different forms [8-11]. Applying external feedback allows students to construct their own internal feedback [12] helping them to evaluate their work and decide if they need further guidance [13], which prompts learners to seek additional feedback iteratively [10, 11]. Moreover, learners must recognize and communicate their learning requirements to refine the feedback process [11]. Hence, learners are expected to utilize feedback they receive, implement strategies to take action on these suggestions, and improve their competencies [6, 10]. Learners who fulfill these roles are considered to be feedback-literate [6, 11].  Student feedback literacy is the ability to interpret and evaluate received feedback and apply it to enhance competencies [6]. Learners’ roles in feedback dialogues are characterized by the features of feedback literacy (Fig 1), reportedly having cognitive, affective, behavioral, and social benefits for their learning. In terms of cognition, feedback literacy helps learners gain knowledge and appreciate the feedback process [14], making learners more likely to engage in feedback activities [15, 16] and coherent in communicating their learning goals with educators [7]. For the affective aspect, learners tend to be more satisfied with the feedback they receive [17] and are more motivated to seek feedback and enact suggestions from these dialogues [16, 18]. Hence, feedback-literate learners display increased confidence in learning from their strengths and weaknesses, thereby efficiently advancing their competencies [18-20]. In addition, learners’ active participation in feedback dialogues would help teachers refine their preparation and delivery of feedback [7]. Therefore, learners’ and teachers’ feedback literacy levels mutually benefit each other’s feedback practices [7]. These tangible benefits of student feedback literacy highlight the importance of maximizing the feedback process utility. |
|  | ii. For thematic analysis, where is the initial coding framework from? Please use table to visualize those codes and references. | We appreciate the insightful feedback. To clarify, our thematic analysis follows an inductive approach where the codes and themes emerge on the data collected. This contrasts with coding based on an initial coding framework – a deductive approach. An inductive approach is adopted because the thematic analysis aims to identify and propose measurable domains of medical student feedback literacy, instead of confirming an existing set of domains.    We have also amended the “Thematic analysis” section to provide a clearer description of the coding and theme development.  **Thematic analysis**  Interview transcripts will be thematically analyzed using NVivo, as this approach is flexible and accessible [47]. As an example, medical students, educators, and patients will be asked the prompt, “Please describe how medical students should receive feedback;” hence, the triangulation of their experiences may provide a comprehensive description of medical student feedback literacy. Codes will be developed based on the collected data [30, 47]. This inductive approach allows for a novel and comprehensive description of medical students’ feedback literacy, that is not confined to existing concepts. The researcher will read the interview transcripts multiple times to become familiar with them. Recurring phrases or keywords will be marked to identify possible patterns in the data and cross-check the patterns that appear among medical students, educators, and patients. Subsequently, codes will be generated to capture notable patterns, which will be checked to reduce overlap and redundancy, and subsequently grouped into potential themes, using an iterative to refine each theme. These themes will be named and clearly defined. Next, these themes will be examined to the entire dataset to ensure that the conclusions made are firmly rooted in the data. The themes will be organized and presented as a thematic map. Additionally, researchers (other than the analyst) will review the themes to enhance the credibility of thematic analysis findings [48]. Finally, the themes will be analyzed to generate measurable domains of medical students’ feedback literacy. |
|  | iii. The benefits of increased feedback literacy can be categorized as cognitive, behavioural, affective/emotional, and social gains. | We appreciate the insightful feedback. We have amended the third paragraph of the introduction section and Fig 1 as per your suggestion.  **Introduction**  Student feedback literacy is the ability to interpret and evaluate received feedback and apply it to enhance competencies [6]. Learners’ roles in feedback dialogues are characterized by the features of feedback literacy (Fig 1), reportedly having cognitive, affective, behavioral, and social benefits for their learning. In terms of cognition, feedback literacy helps learners gain knowledge and appreciate the feedback process [14], making learners more likely to engage in feedback activities [15, 16] and coherent in communicating their learning goals with educators [7]. For the affective aspect, learners tend to be more satisfied with the feedback they receive [17] and are more motivated to seek feedback and enact suggestions from these dialogues [16, 18]. Hence, feedback-literate learners display increased confidence in learning from their strengths and weaknesses, thereby efficiently advancing their competencies [18-20]. In addition, learners’ active participation in feedback dialogues would help teachers refine their preparation and delivery of feedback [7]. Therefore, learners’ and teachers’ feedback literacy levels mutually benefit each other’s feedback practices [7]. These tangible benefits of student feedback literacy highlight the importance of maximizing the feedback process utility.    **Fig 1. Feedback literacy, learners’ roles, and its benefits.** *Abbreviations*: C, Cognitive; A, Affective; B, Behavioural; S, Social. |
|  | iv. I may also invite the authors to be aware of other instrument validation methods, such as Rasch model. | We appreciate the insightful feedback. To clarify, our study protocol follows an instrument validation guide - COSMIN (COnsensus-based Standards for the selection of health Measurement INstruments) which recommends exploratory and confirmatory factor analyses, that ensures construct, convergent and discriminant validities. Thus, Rasch model is not included in our study protocol. We take note of this limitation in our study protocol and have mentioned them in the “Discussion” section.  **DISCUSSION**  Several technical factors must be considered before implementing this protocol. This protocol is being guided by research articles that recommend exploratory and confirmatory factor analyses to establish construct validity [33, 68]. Other methods, such as Rasch analysis, are also appropriate for instrument validation. Similar to other surveys, this study will be subject to a self-reporting bias [46, 85]. The instrument being developed in this study will depend on the reflections and knowledge of feedback literacy among the respondents. This study is also limited by its cross-sectional design, which exposes it to confounding variables [86, 87], such as different learning environments and teacher feedback literacy. Therefore, the next step in this project will be conducting a longitudinal study to assess the development of student feedback literacy. |
