# Supplementary material for: Establishing the measurement and psychometrics of medical student feedback literacy (IMPROVE-FL): A research protocol
Source: PLoS One. 2024 Nov 7;19(11):e0313332. doi: 10.1371/journal.pone.0313332 (PMC11542893; doi:10.1371/journal.pone.0313332)
Supplement: Attachment — Submitted filename: Response to Reviewers R2.docx [file pone.0313332.s003.docx]

| **EDITOR COMMENTS** | |
| --- | --- |
| Comments | Response |
| The manuscript needs proofreading. There are several grammatical errors in the content of the paper. It is suggested to send the paper to a native speaker to do proofreading. | Thank you for your comment. We appreciate your hard work in considering our manuscript and value your comments for improvement.  We have gone through the manuscript and acknowledge that there are grammatical errors in the paper. To solve this, we have sent our paper for a second round of proofreading with Editage. To ensure that the manuscript is grammatically sound and coherent, the authors have also manually gone through the manuscript for a final check.  The edits made to improve the grammar of our manuscript has been highlighted in yellow and can also be reviewed in tracked changes.  We have attached the proofreading certificate for your reference. |
