# Supplementary material for: Establishing the measurement and psychometrics of medical student feedback literacy (IMPROVE-FL): A research protocol
Source: PLoS One. 2024 Nov 7;19(11):e0313332. doi: 10.1371/journal.pone.0313332 (PMC11542893; doi:10.1371/journal.pone.0313332)
Supplement: Attachment — Submitted filename: Response to Reviewers v2.docx [file pone.0313332.s005.docx]

| **EDITOR COMMENTS** | |
| --- | --- |
| Comments | Response |
| Please review your reference list to ensure that it is complete and correct. If you have cited papers that have been retracted, please include the rationale for doing so in the manuscript text or remove these references and replace them with relevant current references. Any changes to the reference list should be mentioned in the rebuttal letter that accompanies your revised manuscript. If you need to cite a retracted article, indicate the article’s retracted status in the References list and also include a citation and full reference for the retraction notice. | Thank you for your reminder. We have double-checked the reference list and found no citations that have been retracted. |
| I appreciate the authors’ meticulous effort in revising the manuscript and thoughtfully addressing the first round of reviewers’ comments. I can see that you have put significant work into enhancing the clarity and depth of the study, which is commendable. Your research, focused on developing and validating a culturally sensitive instrument to measure medical students' feedback literacy, is a valuable and timely contribution to the field of medical education. | We thank the editor for this encouraging comment. |
| However, I have a few minor suggestions to further strengthen your work. The abstract is well-organized, but it would benefit from a clearer articulation of the empirical gap and a stronger emphasis on the relevance of your results, particularly highlighting the instrument’s cross-cultural applicability. | Thank you for your insightful feedback. We have amended the abstract section as per your suggestion.  **Abstract**  Current feedback models advocate learner autonomy in seeking, processing, and responding to feedback so that medical students can become feedback-literate. Feedback literacy improves learners’ motivation, engagement, and satisfaction, which in turn enhance their competencies. However, there is a lack of an objective method of measuring medical student feedback literacy in the empirical literature. Such an instrument is required to determine the level of feedback literacy amongst medical students and whether they would benefit from an intervention. Therefore, this research protocol addresses the methodology aimed at the development of a comprehensive instrument for medical student feedback literacy, which is divided into three phases, beginning with a systematic review. Available instruments in health profession education will be examined to create an interview protocol to define medical students’ feedback literacy from the perspectives of medical students, educators, and patients. A thematic analysis will form the basis for item generation, which will subsequently undergo expert validation and cognitive interviews to establish content validity. Next, we will conduct a national survey to gather evidence of construct validity, internal consistency, hypothesis testing, and test-retest reliability. In the final phase, we will distribute the instrument to other countries in an international survey to assess its cross-cultural validity. This protocol will help develop an instrument that can assist educators in assessing student feedback literacy and evaluating their behavior in terms of managing feedback. Ultimately, educators can identify strengths, and improve communication with students, as well as feedback literacy and the feedback process. In conclusion, this study protocol outlined a systematic, evidence-based methodology to develop a medical student feedback literacy instrument. This study protocol will not only apply to medical and local cultural contexts, but it has the potential for application in other educational disciplines and cross-cultural studies. |
| Additionally, in the introduction, a final statement reinforcing the significance of your study would provide a smoother transition and help frame the next section. | Thank you for your insightful feedback. We have amended the final paragraph of the introduction section as per your suggestion.  **Introduction**  Given the complex nature of feedback literacy, this research protocol proposes a methodology that will lead to the development of a comprehensive instrument for medical student feedback literacy. This protocol will serve as a foundation for several related studies to answer the following research questions:   - What are the available instruments for feedback literacy in healthcare education? - Are these instruments valid and reliable? - How do medical students, medical teachers and patients conceptualize medical student feedback literacy? - What are the measurable domains of medical student feedback literacy? - Are these measurable domains of medical student feedback literacy valid and reliable?   By developing a validated tool to measure feedback literacy, this study aims to fill a critical gap in medical education, ultimately contributing to the improvement of both teaching practices and student outcomes. |
| In the methodology, a bit more clarity in presenting the eligibility criteria and the narrative synthesis approach would improve readability and enhance the methodological rigor. | Thank you for your insightful feedback. We have included the eligibility criteria and further explained our choice of narrative synthesis into the systematic review subsection as per your suggestion.  **Materials and Methods**  **Systematic Review**  Identified studies will be evaluated using the following set of eligibility criteria:   1. included health professions education of any level (undergraduate and/or postgraduate) 2. instrument measuring student feedback literacy or any of its features (regardless of the feedback provider, e.g. peers, patients, teachers) including adaptations, revalidation in a different context and replication studies 3. focusing on students’ role in the feedback process i.e., behaviours and/or attitudes of students towards the feedback process 4. reporting any psychometric properties listed in the Consensus-based Standards for the Selection of Health Measurement Instruments (COSMIN) risk of bias checklist [34] 5. original study published in peer-reviewed journals 6. published in any year   Studies were further excluded if:   1. the instrument does not emphasize feedback literacy or its specific features (such as combination with other unrelated constructs or only mentions feedback literacy briefly) 2. developed instruments that do not focus on the students’ roles in the feedback process, for example, feedback providers opinions on the feedback process 3. non-English publications 4. non-primary research articles, such as reviews, opinion pieces, editorials, or perspectives 5. gray literature (e.g., theses, dissertations, unpublished reports, conference presentations) 6. no available full text   **Materials and Methods**  **Systematic Review**  Findings for the systematic review will be synthesized narratively because it has been successfully used in previous systematic psychometric reviews [35, 36], and it allows analysis between the various conceptualizations of feedback literacy. For example, narrative synthesis allows flexibility to compare between the concepts of Carless and Boud’s feedback literacy [10] and the Bing-You et al. FEEDME Culture [37]. Thus, narrative synthesis will be used for its inclusiveness, unlike other synthesis approach such as meta-analysis, which restricts a synthesis to one single concept used in all studies included. The narrative synthesis will be guided by Popay et al.’s [38] recommendations. |
| A brief explanation for choosing the 7-point scale over other options would also add value, especially in justifying the sensitivity and appropriateness of the scale for this study’s context. | Thank you for your insightful feedback. We have further explained our decision to use a 7-point semantic differential scale in the item generation subsection. Based on our understanding of the literature, there is no one absolute right answer, but depending on the rationales and school of thoughts.  **Materials and Methods**  **Item Generation**  Participants will be asked to rate their responses using a 7-point semantic differential scale ranging from 1 (do not correspond at all) to 7 (correspond exactly). This 7-point semantic differential scale is chosen to improve the reliability of the instrument (as opposed to a 5 or 6-point scale) [54] while maintaining conciseness (as opposed to an 8, 9 or 10-point scale) [42], and allow a participant to express a true neutral stance (as opposed to an even-numbered scale) [42]. The Flesch-Kincaid reading ease score will be examined to ensure the items are clear and concise for easier interpretation [55, 56]. |
| Lastly, while your discussion section is comprehensive, I recommend adding a separate conclusion and recommendation section. This will provide a clear and impactful summary of your study’s key contributions and its practical implications for future research and educational interventions. | Thank you for your insightful feedback. We have added a conclusion section to conclude the manuscript and suggest future recommendations.  **Conclusion**  In conclusion, this study protocol outlined a systematic, evidence-based methodology to develop a medical student feedback literacy instrument. Careful consideration was given into the future development of this instrument so that it will have good internal consistency, test-retest reliability, content validity, construct validity, hypotheses testing and cross-cultural validity. The methodology outlined here does not only serve medical and local cultural contexts, but it has the potential for application in other educational disciplines and cross-cultural studies. Moving forward, researchers can use this study protocol as a guide to develop instruments, measure, and design effective interventions for student feedback literacy. |

| **REVIEWER #2 COMMENTS** | | |
| --- | --- | --- |
| Q | Comments | Response |
| 1. | *Does the manuscript provide a valid rationale for the proposed study, with clearly identified and justified research questions?*  **Yes.** | We thank the reviewer for all their comments. We appreciate your efforts in providing feedback to our manuscript and helping us to improve on it. |
| 2. | *Is the protocol technically sound and planned in a manner that will lead to a meaningful outcome and allow testing the stated hypotheses?*  **Yes.** | We thank the reviewer for their encouraging comment. |
| 3. | *Is the methodology feasible and described in sufficient detail to allow the work to be replicable?*  **Yes.** | We thank the reviewer for their encouraging comment. |
| 4. | *Have the authors described where all data underlying the findings will be made available when the study is complete?*  **Yes.** | We thank the reviewer for their encouraging comment. |
| 5. | *Is the manuscript presented in an intelligible fashion and written in standard English?*  **Yes.** | We thank the reviewer for their encouraging comment. |
| 6. | i. I want to suggest this title: "Establishing the Measurement and Psychometrics of Medical Student Feedback Literacy (IMPROVE-FL): A Research Protocol. | Thank you for your comment. We agree that your title suggestion represents the whole manuscript better and have amended it accordingly.  Establishing the Measurement and Psychometrics of Medical Student Feedback Literacy (IMPROVE-FL): A Research Protocol. |
|  | ii. The aspect using test-retest after internal consistency method of reliability of the instruments should be revisited. | Thank you for your insightful feedback.  If you are concerned regarding the use of both internal consistency and test-retest, we would like to clarify that although they are both tests for reliability, they cover different types of reliability. According to Creswell and Creswell (2018), internal consistency is used to measure the homogeneity of items (and the response given by users) throughout a set of instrument items. Meanwhile, test-retest is used to measure the stability of sample scores from one administration to another. Additionally, both these tests have been recommended by the COSMIN guideline to reduce risk of bias in an instrument (Mokkink et al., 2018). The purpose of these two reliability tests have been described in the respective sub-headings.  **Internal consistency**  Based on the factors produced via the EFA, the internal consistency between these factors will be determined to evaluate whether the scores are consistent throughout the instrument [30].  **Test-retest reliability**  Test-retest reliability will be evaluated to examine the stability of our measurements over time [30].  If you are concerned regarding the sequence of the two reliability tests, we would like to clarify that the tests will be done simultaneously after the national survey, during data analysis. Therefore, the analysis or the sequence of it will bear little to no effect on the outcome.  We hope our response addresses your concern. Please let us know if you have other concerns not addressed in this document. |
|  | iii. I think the research questions / hypothesis of the study should have been stated clearly under a section like the introduction. | Thank you for your insightful feedback. We have amended the introduction section as per your suggestion.  **Introduction**  Given the complex nature of feedback literacy, this research protocol proposes a methodology that will lead to the development of a comprehensive instrument for medical student feedback literacy. This protocol will serve as a foundation for several related studies to answer the following research questions:   - What are the available instruments for feedback literacy in healthcare education? - Are these instruments valid and reliable? - How do medical students, medical teachers and patients conceptualize medical student feedback literacy? - What are the measurable domains of medical student feedback literacy? - Are these measurable domains of medical student feedback literacy valid and reliable?   By developing a validated tool to measure feedback literacy, this study aims to fill a critical gap in medical education, ultimately contributing to the improvement of both teaching practices and student outcomes. |

| **REVIEWER #3 COMMENTS** | | |
| --- | --- | --- |
| Q | Comments | Response |
| 1. | *Does the manuscript provide a valid rationale for the proposed study, with clearly identified and justified research questions?*  **Yes.** | We thank the reviewer for all their comments. We appreciate your efforts in providing feedback to our manuscript and helping us to improve on it. |
| 2. | *Is the protocol technically sound and planned in a manner that will lead to a meaningful outcome and allow testing the stated hypotheses?*  **Yes.** | We thank the reviewer for their encouraging comment. |
| 3. | *Is the methodology feasible and described in sufficient detail to allow the work to be replicable?*  **Yes.** | We thank the reviewer for their encouraging comment. |
| 4. | *Have the authors described where all data underlying the findings will be made available when the study is complete?*  **Yes.** | We thank the reviewer for their encouraging comment. |
| 5. | *Is the manuscript presented in an intelligible fashion and written in standard English?*  **Yes.** | We thank the reviewer for their encouraging comment. |
| 6.1 | *Abstract and keywords:* The abstract is organized neatly in IMRD style. The keywords are appropriate for the study's topic. However, rather than saying "a gap exists," explain why this gap matters and be more explicit about the existing gap in the literature (line 24-25).  Also, the relevance and wider results might be emphasized by the author / authors. You want readers to comprehend the procedure as well as the significance of it.  In addition, emphasize how your technique is different from other instruments, particularly with regard to its cross-cultural validity. Make sure the whole abstract is well summarized. | Thank you for your insightful feedback. We have amended the abstract section as per your suggestion.  **Abstract**  Current feedback models advocate learner autonomy in seeking, processing, and responding to feedback so that medical students can become feedback-literate. Feedback literacy improves learners’ motivation, engagement, and satisfaction, which in turn enhance their competencies. However, there is a lack of an objective method of measuring medical student feedback literacy in the empirical literature. Such an instrument is required to determine the level of feedback literacy amongst medical students and whether they would benefit from an intervention. Therefore, this research protocol addresses the methodology aimed at the development of a comprehensive instrument for medical student feedback literacy, which is divided into three phases, beginning with a systematic review. Available instruments in health profession education will be examined to create an interview protocol to define medical students’ feedback literacy from the perspectives of medical students, educators, and patients. A thematic analysis will form the basis for item generation, which will subsequently undergo expert validation and cognitive interviews to establish content validity. Next, we will conduct a national survey to gather evidence of construct validity, internal consistency, hypothesis testing, and test-retest reliability. In the final phase, we will distribute the instrument to other countries in an international survey to assess its cross-cultural validity. This protocol will help develop an instrument that can assist educators in assessing student feedback literacy and evaluating their behavior in terms of managing feedback. Ultimately, educators can identify strengths, and improve communication with students, as well as feedback literacy and the feedback process. In conclusion, this study protocol outlined a systematic, evidence-based methodology to develop a medical student feedback literacy instrument. This study protocol will not only apply to medical and local cultural contexts, but it has the potential for application in other educational disciplines and cross-cultural studies. |
| 6.2 | *Introduction*: The introduction provides a solid background on the importance of feedback literacy, the gap in the empirical literature, and the specific context of medical education. At line 44, after mentioning formative assessments, you could add this: “Feedback is especially important in medical education as it affects patient safety and clinical competence in addition to scholastic performance. Preparing well-rounded, capable healthcare practitioners so becomes dependent on developing feedback literacy." | Thank you for your insightful feedback. We have added the sentences to the introduction section as per your suggestion. To maintain a seamless transition, the last sentence of the first paragraph has been merged with the first sentence of the second paragraph.  **Introduction**  The theory of feedback emerged as a form of behaviorism, in which behaviors are reinforced to inform learners [1]. Feedback is an integral part of formative assessments that are a strategy adopted by teachers to direct student learning [2]. Feedback is especially important in medical education as it affects patient safety [3, 4] and clinical competence [5] in addition to scholastic performance [5, 6]. Preparing well-rounded, capable healthcare practitioners so becomes dependent on developing feedback literacy.  The current concept of feedback has since transformed from a unidirectional tool to one involving students’ bidirectional engagement for holistic learning [7], which facilitates focused feedback models that target specific cognitive processes [8], student perspectives [9], and the pedagogical aspects of feedback [10]. Accordingly, learners must receive feedback in a positive manner, and teachers need to formulate and deliver feedback efficiently [11]. This shared responsibility motivates students to seek, process, and respond to feedback from multiple sources and different forms [12-15]. Applying external feedback allows them to construct their own internal feedback [16], thereby helping to evaluate their work and decide if they need further guidance [17], which prompts them to seek additional feedback iteratively [14, 15]. Moreover, to refine the feedback process, learners must recognize and communicate their learning requirements [15]. Hence, they are expected to utilize feedback, implement strategies to take action on these suggestions, and improve their competencies [10, 14]. Learners who fulfill these roles are considered feedback-literate [10, 15]. |
| 6.3 | *Introduction*: Also, please be more explicit about the empirical gap you mentioned. At line 79-80, the author(s) could be more explicit and show the novelty of this research. To provide a seamless transition to the following part, include a last statement in the introduction. This may also serve to emphasize the importance of your study. Ex. "By developing a validated tool to measure feedback literacy, this study aims to fill a critical gap in medical education, ultimately contributing to the improvement of both teaching practices and student outcomes." | Thank you for your insightful feedback. We have amended the paragraph 4, 5, and 6 of the introduction section as per your suggestion.  **Introduction**  Measuring feedback literacy can help educators identify areas of strength and improvement to promote the development of feedback-literate learners. Such an instrument would be invaluable in assessing the cycle illustrated in Fig. 1 and identifying the specific features and activities with which learners require help. However, a recent scoping review reports that available feedback literacy instruments require further evaluation and validation [23]. Therefore, there is a current need for a valid instrument to be developed based on empirical findings to allow a comprehensive measurement of student feedback literacy. Consequently, interventions for student feedback literacy can be objectively evaluated and compared. In short, a reliable and valid instrument is required to measure student feedback literacy.  When developing a comprehensive instrument for measuring such literacy, several factors must be considered. First, developing such an instrument should include the learners’ perspectives as they are integral to effective feedback [10, 15]. However, instruments should be contextualized in the mode of feedback delivery [24]. For example, medical students’ feedback literacy should be specialized because it involves patient safety [3] and active workplace teaching and learning [25, 26]. By comparison, feedback in language education focuses on written corrective feedback [27, 28], which may not be applicable in the medical education context. Culture also plays a role in feedback practices [29, 30]; hence, the instrument can be examined for both accessibility and inclusiveness. Thus, the methodology for developing a feedback literacy measurement instrument should consider learner perspectives, match the educational or training context, and be culturally sensitive.  Given the complex nature of feedback literacy, this research protocol proposes a methodology that will lead to the development of a comprehensive instrument for medical student feedback literacy. This protocol will serve as a foundation for several related studies to answer the following research questions:   - What are the available instruments for feedback literacy in healthcare education? - Are these instruments valid and reliable? - How do medical students, medical teachers and patients conceptualize medical student feedback literacy? - What are the measurable domains of medical student feedback literacy? - Are these measurable domains of medical student feedback literacy valid and reliable?   By developing a validated tool to measure feedback literacy, this study aims to fill a critical gap in medical education, ultimately contributing to the improvement of both teaching practices and student outcomes. |
| 6.4 | *Methodology*: The methods is well-organized, but here are a few suggestions to improve this manuscript:   a) Move the mention of these criteria (lines 124–125) to an earlier position in the paragraph or make them clearer by presenting them in bullet points or a table. | Thank you for your insightful feedback. We have included the eligibility criteria into the systematic review subsection as per your suggestion.  **Materials and Methods**  **Systematic Review**  Identified studies will be evaluated using the following set of eligibility criteria:   1. included health professions education of any level (undergraduate and/or postgraduate) 2. instrument measuring student feedback literacy or any of its features (regardless of the feedback provider, e.g. peers, patients, teachers) including adaptations, revalidation in a different context and replication studies 3. focusing on students’ role in the feedback process i.e., behaviours and/or attitudes of students towards the feedback process 4. reporting any psychometric properties listed in the Consensus-based Standards for the Selection of Health Measurement Instruments (COSMIN) risk of bias checklist [34] 5. original study published in peer-reviewed journals 6. published in any year   Studies were further excluded if:   1. the instrument does not emphasize feedback literacy or its specific features (such as combination with other unrelated constructs or only mentions feedback literacy briefly) 2. developed instruments that do not focus on the students’ roles in the feedback process, for example, feedback providers opinions on the feedback process 3. non-English publications 4. non-primary research articles, such as reviews, opinion pieces, editorials, or perspectives 5. gray literature (e.g., theses, dissertations, unpublished reports, conference presentations) 6. no available full text |
|  | b) Instead of simply stating that the search string will be in Appendix S1, a brief overview of the string would help readers understand the comprehensiveness of the search strategy (e.g., "including terms related to feedback, medical students, psychometrics"). | Thank you for your insightful feedback. We have included a brief overview of the search string in the first sentence of paragraph 2 of the systematic review subsection. However, to improve clarity we have rearranged the sentences in this paragraph and amended them.  **Materials and Methods**  **Systematic Review**  Initially, a pilot search will be performed to select keywords that return relevant research articles. Based on the PICO (Population-Intervention-Comparison-Outcome) format, terms and synonyms related to healthcare education students, feedback literacy, psychometric properties, and instruments will be employed to perform a systematic search of six electronic databases (i.e., Scopus, Medline, Web of Science, CINAHL Complete, Education Research Complete, and Psychology and Behavioral Sciences Collection). The full search string for each database is provided in Appendix S1. The references and citations of these articles will be manually screened for additional potential articles [32, 33]. Periodic searches will be conducted to identify newly published studies and ensure that the review accounts for all recent publications. |
|  | c) Provide a bit more detail about what specific risks you will be focusing on using COSMIN. | Thank you for your insightful feedback. We have included some specific psychometric properties based on the COSMIN checklist in paragraph 3 of the systematic review subsection.  **Materials and Methods**  **Systematic Review**  After removing duplicates, two authors will independently screen the retrieved records based on their titles, abstracts, and keywords. Subsequently, the full texts will be screened. Any differing opinions will be resolved through discussions between the authors. Consequently, relevant data, such as the description, utility, constructs, and psychometrics of each included instrument, will be extracted. The methodological quality of the studies will be independently appraised by two authors according to the COSMIN Risk of Bias Checklist [34]. This checklist was selected because of its comprehensive and robust methodology applied in its development. It covers a wide range of psychometric properties thoroughly, including content validity, structural validity, hypotheses testing, cross-cultural validity, internal consistency and test-retest reliability. |
|  | d) Consider including a brief explanation of why you chose narrative synthesis and how it compares to other synthesis methods, especially in the context of psychometric reviews. | Thank you for your insightful feedback. We have further clarified our choice of synthesis approach in paragraph 3 of the systematic review subsection.  **Materials and Methods**  **Systematic Review**  Findings for the systematic review will be synthesized narratively because it has been successfully used in previous systematic psychometric reviews [35, 36], and it allows analysis between the various conceptualizations of feedback literacy. For example, narrative synthesis allows flexibility to compare between the concepts of Carless and Boud’s feedback literacy [10] and the Bing-You et al. FEEDME Culture [37]. Thus, narrative synthesis will be used for its inclusiveness, unlike other synthesis approach such as meta-analysis, which restricts a synthesis to one single concept used in all studies included. The narrative synthesis will be guided by Popay et al.’s [38] recommendations. |
|  | e) You refer to "maximum variation sampling" (line 173), which is excellent for qualitative research; nonetheless, please include a brief explanation of the standards for background-based participant selection. | Thank you for your insightful feedback. We have further clarified our sampling strategy with an example, in paragraph 3 of the “interviews with medical students, medical educators, and patients” subsection.  **Materials and Methods**  **Interviews with medical students, medical educators, and patients**  To ensure the quick and wide reach of invitations, interviewees will be recruited via social media posts and student societies. The interviews will include medical students studying in Malaysia, medical educators responsible for providing feedback, and patients with experience in providing feedback to medical students over the past month to reduce the risk of recall bias [48]. Data collection will cease after reaching a point of saturation at which no additional data can be retrieved. The expected number of interviews will include 10 focus group discussions (ideally including four medical students in one group; n = 40), 20 medical educators, and 20 patients or caretakers. Interviewees from various backgrounds will be identified and approached (e.g., ethnicity, year of study/teaching, department, public and private medical school, inpatient, or outpatient) to achieve maximum variation sampling [31]. For example, if the first interviewee is a Malay, Year 3 medical student from Universiti Malaya doing their orthopaedics rotation, the next interviewee would ideally be a Chinese, Year 5 medical student from Universiti Tunku Abdul Rahman, doing their internal medicine rotation. Interviewees will be recruited to include a most diverse backgrounds possible to achieve the standard of maximum variation sampling. |
|  | f) You refer to pilot testing in lines 164 and 244. To show flexibility and adaptability, it might be useful to make clear how any modifications from the pilot would be included into the main research design. | Thank you for your insightful feedback. We have further clarified how changes from the pilot tests will be applied to the interviews and cognitive interviews, in their respective subsections.  **Materials and Methods**  **Interviews with medical students, medical educators, and patients**  An interview protocol was designed based on the findings of our review of student feedback literacy conceptualizations [10, 15] and Merriam and Tisdell’s four types of good interview questions [47]. For example, “Ideally, what do you think a good medical student should do to make the most of the feedback they receive?” can be used as an “ideal position” question to gather opinions on the feedback literacy feature “taking actions.” The interview protocol will be piloted to ensure comprehensiveness and comprehensibility [47]. Any modifications deemed necessary from the pilot test will be applied to the interview protocol to allow flexibility and adaptability. For example, interview questions might need to be revised to include examples to elicit elaborate and accurate responses from the interviewees.  **Cognitive Interviews**  An interview protocol will be designed based on the drafted items and piloted with two to three medical students to test its feasibility and implementation of the interview protocol [59]. The cognitive interview protocol might be revised after the pilot test to ensure that the questions elicit the needed response from medical students regarding the comprehensibility of the questionnaire items. During the interviews, the think-aloud approach will be adopted to ask interviewees to verbalize their thought processes while answering the questionnaires [60]. In addition, probing questions will be used to guide the focus on specific parts and ensure clarity among the interviewees [61]. Interview transcripts will be analyzed to identify areas for improvement and the questionnaire modified based on these findings. The cognitive interviews will be repeated until no new problems are identified and the questionnaire is deemed comprehensible and comprehensive [61]. |
|  | g) It may be useful to clarify if intercoder reliability metrics like Cohen's kappa will be used and the number of researchers who will be participating in the coding process when you explain the coding procedure (line 187). This would show rigor. | Thank you for your insightful feedback. To clarify, it is planned that the coding process will be done by one researcher. Later, the codes and themes will be reviewed by at least two other researchers (separate from the coder) to ensure credibility. We have amended the thematic analysis subsection to further clarify this planned procedure.  For qualitative analysis, review by different researchers is more applicable than interrater reliability metrics such as Cohen’s kappa because our analysis does not involve ratings (e.g., 1, 2, 3, etc).  **Materials and Methods**  **Thematic Analysis**  Interview transcripts will be thematically analyzed using NVivo as this approach is flexible and accessible [49]. As an example, medical students, educators, and patients will be asked the prompt, “Please describe how medical students should receive feedback.” Accordingly, triangulating their experiences may provide a comprehensive description of medical student feedback literacy. Codes will be developed based on the collected data [31, 49] by one researcher. This inductive approach allows for a novel and comprehensive description of medical students’ feedback literacy that is not confined to existing concepts. This researcher will read the interview transcripts multiple times to become familiar with them. Recurring phrases or keywords will be highlighted to identify possible patterns in the data and cross-check those that appear among medical students, educators, and patients. Subsequently, codes will be generated to capture notable patterns, which will be checked to reduce overlap and redundancy, and subsequently grouped into potential themes using an iterative approach to refine each theme. These themes will be named and clearly defined. Next, these will be examined across the entire dataset to ensure that the conclusions drawn are firmly rooted in the data. The themes will be organized and presented as a thematic map. Additionally, at least two researchers (other than the coder) will review the themes to enhance the credibility of the findings [50]. Finally, the themes will be analyzed to generate measurable domains of medical students’ feedback literacy. |
|  | h) Line 183 mentions triangulating patients, teachers, and students. Indicate if source triangulation is something you intend to do. | Thank you for your insightful feedback. We do intend for a source triangulation and have amended the thematic analysis subsection to improve clarity.  **Materials and Methods**  **Thematic Analysis**  Interview transcripts will be thematically analyzed using NVivo as this approach is flexible and accessible [49]. As an example, medical students, educators, and patients will be asked the prompt, “Please describe how medical students should receive feedback.” Accordingly, triangulating the different experiences between three types of interviewees (source triangulation) may provide a comprehensive description of medical student feedback literacy. Codes will be developed based on the collected data [31, 49] by one researcher. This inductive approach allows for a novel and comprehensive description of medical students’ feedback literacy that is not confined to existing concepts. This researcher will read the interview transcripts multiple times to become familiar with them. Recurring phrases or keywords will be highlighted to identify possible patterns in the data and cross-check those that appear among medical students, educators, and patients. Subsequently, codes will be generated to capture notable patterns, which will be checked to reduce overlap and redundancy, and subsequently grouped into potential themes using an iterative approach to refine each theme. These themes will be named and clearly defined. Next, these will be examined across the entire dataset to ensure that the conclusions drawn are firmly rooted in the data. The themes will be organized and presented as a thematic map. Additionally, at least two researchers (other than the coder) will review the themes to enhance the credibility of the findings [50]. Finally, the themes will be analyzed to generate measurable domains of medical students’ feedback literacy. |
| 6.5 | *Instruments*: Explain the decision to use a 7-point scale rather than a 5- or 10-point one (line 213). | Thank you for your insightful feedback. We have further explained our decision to use a 7-point semantic differential scale in the item generation subsection. Based on our understanding of the literature, there is no one absolute right answer, but depending on the rationales and school of thoughts.  **Materials and Methods**  **Item Generation**  Participants will be asked to rate their responses using a 7-point semantic differential scale ranging from 1 (do not correspond at all) to 7 (correspond exactly). This 7-point semantic differential scale is chosen to improve the reliability of the instrument (as opposed to a 5 or 6-point scale) [54] while maintaining conciseness (as opposed to an 8, 9 or 10-point scale) [42], and allow a participant to express a true neutral stance (as opposed to an even-numbered scale) [42]. The Flesch-Kincaid reading ease score will be examined to ensure the items are clear and concise for easier interpretation [55, 56]. |
|  | Was the population of medical students subjected to pilot testing to ensure readability and usability? | Thank you for your insightful feedback. For your information, our team did consider a pilot test prior to the national survey. In several past studies, cognitive interviews are recognised as a form of pilot study but conducting in a qualitative manner (Wildy & Clarke, 2009).  Wildy, H., Clarke, S. Using cognitive interviews to pilot an international survey of principal preparation: A Western Australian perspective. Educ Asse Eval Acc 21, 105–117 (2009). https://doi.org/10.1007/s11092-009-9073-3  However, we have chosen the term “cognitive interviews” over pilot test. A cognitive interview can assess whether an instrument is comprehensible, readable and usable.    According to the COSMIN guidelines, content validity (including the comprehensibility, readability and usability) of an instrument can be assessed by either (qualitative) cognitive interview or (quantitative) pilot testing. Since there is no requirement for both procedures and because we prefer to explore the reasons why a student might find our instrument to be lacking in comprehensibility, we have chosen cognitive interviews.  To ensure that our choice is made clear in the manuscript, the cognitive interviews subsection has been amended.  **Materials and Methods**  **Cognitive Interviews**  The first draft of the questionnaire will be validated by experts and then tested with a group of medical students to strengthen comprehensibility. Cognitive interviews allow the exploration of the reasons why medical students might find the questionnaire to be comprehensible or lack thereof. The execution of cognitive interviews will follow the recommendations of Scott et al., who provide a step-by-step approach with illustrative examples [58]. Semi-structured individual interviews will be conducted with Malaysian medical students, who are the target population of the survey, face-to-face or virtually, and a researcher will be trained to conduct them. The interviews will be audiotaped and then transcribed verbatim. |
| 6.6 | *Discussion*: The discussion section is in order. Well captured. However, I feel there should also be a separate section for conclusion and recommendation. This is very important for a robust work. | Thank you for your insightful feedback. We have added a conclusion section to conclude the manuscript and suggest future recommendations.  **Conclusion**  In conclusion, this study protocol outlined a systematic, evidence-based methodology to develop a medical student feedback literacy instrument. Careful consideration was given into the future development of this instrument so that it will have good internal consistency, test-retest reliability, content validity, construct validity, hypotheses testing and cross-cultural validity. The methodology outlined here does not only serve medical and local cultural contexts, but it has the potential for application in other educational disciplines and cross-cultural studies. Moving forward, researchers can use this study protocol as a guide to develop instruments, measure, and design effective interventions for student feedback literacy. |

References

Creswell, J. W., & Creswell, J. D. (2018). *Research design: Qualitative, quantitative, and mixed methods approaches* (5th ed.). Sage.

Mokkink, L. B., De Vet, H. C. W., Prinsen, C. A. C., Patrick, D. L., Alonso, J., Bouter, L. M., & Terwee, C. B. (2018). COSMIN risk of bias checklist for systematic reviews of patient-reported outcome measures. *Quality of Life Research, 27*, 1171-1179. <https://doi.org/doi>: 10.1007/s11136-017-1765-4
