# Supplementary material for: Establishing the measurement and psychometrics of medical student feedback literacy (IMPROVE-FL): A research protocol
Source: PLoS One. 2024 Nov 7;19(11):e0313332. doi: 10.1371/journal.pone.0313332 (PMC11542893; doi:10.1371/journal.pone.0313332)
Supplement: Attachment — Submitted filename: Reviewer Comments - PONE-D-23-27190_R2.docx [file pone.0313332.s004.docx]

The research is an exploratory sequential mixed-methods study aimed at investigating medical students' feedback literacy, with the ultimate goal of developing and validating a new instrument to measure it. It appears to me that the purpose is to develop an innovative, validated, culturally sensitive method for evaluating medical students' feedback literacy that may be utilized for educational interventions and future research.

The author(s) have done a good job addressing other reviewers’ comments. But, after a detailed reading of the article, here is my review of the article:

1. Abstract and keywords: The abstract is organized neatly in IMRD style. The keywords are appropriate for the study's topic. However, rather than saying "a gap exists," explain why this gap matters and be more explicit about the existing gap in the literature (line 24-25). Also, the relevance and wider results might be emphasized by the author/ authors. You want readers to comprehend the procedure as well as the significance of it. In addition, emphasize how your technique is different from other instruments, particularly with regard to its cross-cultural validity. Make sure the whole abstract is well summarized.
2. Introduction: The introduction provides a solid background on the importance of feedback literacy, the gap in the empirical literature, and the specific context of medical education. At line 44, after mentioning formative assessments, you could add this: Feedback is especially important in medical education as it affects patient safety and clinical competence in addition to scholastic performance. Preparing well-rounded, capable healthcare practitioners so becomes dependent on developing feedback literacy."
3. Also, please be more explicit about the empirical gap you mentioned. At line 79-80, the author(s) could be more explicit and show the novelty of this research. To provide a seamless transition to the following part, include a last statement in the introduction. This may also serve to emphasize the importance of your study. Ex. "By developing a validated tool to measure feedback literacy, this study aims to fill a critical gap in medical education, ultimately contributing to the improvement of both teaching practices and student outcomes."
4. Methodology: The methods is well-organized, but here are a few suggestions to improve this manuscript:
a) Move the mention of these criteria (lines 124–125) to an earlier position in the paragraph or make them clearer by presenting them in bullet points or a table.
b) Instead of simply stating that the search string will be in Appendix S1, a brief overview of the string would help readers understand the comprehensiveness of the search strategy (e.g., "including terms related to feedback, medical students, psychometrics").
c) Provide a bit more detail about what specific risks you will be focusing on using COSMIN.
d) Consider including a brief explanation of why you chose narrative synthesis and how it compares to other synthesis methods, especially in the context of psychometric reviews.
e) You refer to "maximum variation sampling" (line 173), which is excellent for qualitative research; nonetheless, please include a brief explanation of the standards for background-based participant selection.
f) You refer to pilot testing in lines 164 and 244. To show flexibility and adaptability, it might be useful to make clear how any modifications from the pilot would be included into the main research design.
g) It may be useful to clarify if intercoder reliability metrics like Cohen's kappa will be used and the number of researchers who will be participating in the coding process when you explain the coding procedure (line 187). This would show rigor.
h) Line 183 mentions triangulating patients, teachers, and students. Indicate if source triangulation is something you intend to do.
5. Instruments: Explain the decision to use a 7-point scale rather than a 5- or 10-point one (line 213). Was the population of medical students subjected to pilot testing to ensure readability and usability?
6. Discussion: The discussion section is in order. Well captured. However, I feel there should also be a separate section for conclusion and recommendation. This is very important for a robust work.
